# The Customs and Diseases of the New Zealanders

**Published:** 1855-07

**Authors:** Arthur S. Thompson


THE CUSTOMS AND DISEASES OF THE NEW ZEALANDERS. By ARTHUR S. THOMPSON, M. D. *Cancer.*—From very careful inquiries I have not heard of a native woman dying from cancer or carcinoma of the breast. If the disease should occur, it must be extremely rare indeed. This remarkable peculiarity deserves particular attention; but the absence of the disease from among the New Zealanders is another link in the chain of evidence that cancer is a disease of civilization. Sir Astley Cooper recommends a diet of animal food as a means of cure in cancer; but the absence of the disease among a people who live almost entirely on vegetables, would indicate a different opinion. As cancer is said to be rare among the inhabitants of Egypt and Algeria, a migration to these countries has been recommended to those suffering under the malady. The same may be said of New Zealand. But I have already said that, although the aborigines do not suffer from it, yet European women have had the disease commence in the country; and there are no European women sufficiently old, who have been born and brought up in New Zealand, so as to ascertain whether they are liable to disease or not.

*Stone in the Bladder.*—No case of this disease has been seen, nor have I heard of one. As Prof. Cooper has calculated that one case occurs annually in England for every hundred thousand souls, we might naturally expect, out of a population supposed to be nearly one hundred thousand, that ten years' constant intercourse would have brought one instance of stone under the notice of some of the Europeans living in the country, had the disease existed among the New Zealanders. The vegetable diet, and the large quantity of pure water which the New Zealanders drink, tend to produce urine of a low specific gravity, and comparatively free from uric acid.

*Bronchocele.*—I heard of one supposed case of this disease, but on inquiry found it to be scrofulous tumor. There is in the North Island of New Zealand, in which almost all the native population live, a large quantity of magnesian limestone, which cannot fail to impregnate the water running through it; but I have visited villages in this part of the country, but never saw a case of bronchocele. This circumstance furnishes no argument against the theory of the magnesian origin of bronchocele, because the aborigines rarely use well water, and they are very particular that the river water they use is clear and tasteless. If, then, there is one thing more than another the New Zealanders are particular about, it is the purity of the water they drink. Thus, well water is rarely used, or a stream which runs through a wood, where decayed trees are likely to be found.

*Hydrophobia* has not been seen either among dogs, Europeans, or natives. The absence of the disease in New Zealand is a link in the chain of evidence that the malady has its origin in specific contagion. There are many dogs in the country, and some of them are very badly fed animals.

*Tetanus.*—I have not seen nor heard of either an idiopathic or symptomatic case of tetanus among the aborigines.

*Diseases of Infancy.*—A large number of children die under three years of age. The poor diet on which the mother lives produces thin and watery milk; sometimes the milk is scanty, and food is given to the child which it cannot digest. This produces bowel complaints and fever. Neglected catarrhal complaints from insufficient clothing often produce death. Dentition is occasionally accompanied with irritation and convulsions; the last complaint is not so common as among European children; croup proves fatal; worms are very frequent. Tabes mesenterica occurs, but the poor food infants have given to them lays the foundation of a delicate, sickly life, and a premature old age.

*Diseases of the Organs of Menstruation.*—As there are a large number of women in New Zealand barren, it may be supposed the menstrual discharge is often irregular or diseased. I have inquired into this subject, but it is a difficult question to arrive at the truth of, because the native women rarely consult Europeans about this class of diseases. From the inquiries I have made, it appears the menses begin to flow at thirteen, fourteen, fifteen, or sixteen. I have heard it stated they begin at ten years of age, but this is not usual. I never saw a New Zealand girl have a child who, in appearance, was not at least fifteen years of age. I have seen European mothers younger. Sexual intercourse takes place often before the menses appear. The menses are sometimes very irregular, one or two months often passing without a flow; women occasionally have headache for a day or two before the usual period of the menses, but no uneasiness is felt after this. I have heard of some instances of menorrhagia; profuse menstruation occurs. From all inquiries, I am of opinion that the New Zealand women are subject to the same irregularities as women in England; but these irregularities are perhaps not so common, nor do they appear to have so much influence on the constitution. This opinion is founded, however, on no statistical data. Hysteria and chlorosis, the two maladies most influenced by the menses, are almost unknown. I have heard of a half-caste girl who had chlorosis. The sterility which the New Zealand women suffer from, is perhaps caused by too early and promiscuous sexual intercourse. I have been told that women have a way of rendering themselves barren by injuring the womb, but as my informant, one of the clergy of the Church Missionary Society, said it was never attended with loss of life, I am inclined to doubt it. Count Strezlecti in his "Physical History of New South Wales," published in 1845, states that when once a female of the aboriginal tribes of Australia has borne a child to a European or white, she ceases entirely to reproduce with males of her own race. I doubt the truth of this among the aborigines of Australia, and can deny its accuracy regarding the New Zealand women, for I know several instances of New Zealand women having children with men of their own race, after having had children by Europeans.

*Parturition* is not attended with the same dangers as among Europeans. Many missionaries and medical men never heard of a women dying in childbed. A native chief, aged about fifty, told me that out of a tribe numbering four thousand souls, he could only recollect ten instances of women dying in childbed. This is about one death in three years out of two thousand women. The circumstances which caused death, the chief said, were hemorrhage and cross-births; one medical man was called to a woman with an arm presentation, and another to a protracted labor, the result of a deformed pelvis. Child-bearing extends from fifteen to thirty-five years of age; but I heard of a woman whose age, from certain known circumstances, must have been forty-seven when she gave birth to a child.

New Zealand women often give birth to large families. Twins and triplets occur; but three children born at one time have never been reared. I knew a women who had fifteen children, all dead. When a woman has a protracted labor, it is assisted by violent pressure on the abdomen. I saw a young female who was suffering from extensive ulceration of the muscles of the abdomen, which had come on after a protracted labor. It might have been produced by too violent pressure. Abortions occur, and are often caused and produced by pressure on the abdomen, or sitting over a native oven, such as will be afterwards described. I never heard of a woman with puerperal convulsions. Infanticide is generally perpetrated the moment the child is born, by pressing the head between the thighs.

Child-bearing is usually easy with New Zealanders. A New Zealand woman, the bearer of a burden, with a party of travelers, was confined on the road; after the birth of the child she walked four miles, and next day fifteen. They rise almost immedately after the expulsion of the after-birth. As soon as a New Zealand woman finds her labor about to commence, she takes a blanket and goes out into the open air, into a quiet, retired place. If it is her first child, a woman attends her: after the first child they go out alone. After delivery the woman proceeds to a stream and washes herself and infant, and then returns home. During labor the women kneel down, with their thighs apart, and having their hands resting on a tree or stick. They hold their breath. Labor seldom exceeds two hours; generally it is much shorter. Sickness after parturition is rare. The great ease of childbirth may be partly due to the pyramidal shape of the skull. Prolapsus uteri after childbirth is rare. In a large village containing four hundred women there was only one woman who had this disease.

*On the method in which Diseases were treated by the New Zealanders.*—The instinct to live, which is found so strong in the human breast, has made men, in all ages, endeavor to procure means to ward off death. Among the New Zealanders the desire to live was as strong as among other races of men; yet the idea which existed in their minds, that all diseases are inflicted, directly or indirectly, by the gods for their sins, or by witchcraft, made them resort to prayers in place of physic for a restoration to health. I insert four out of the many prayers which were used for the treatment of disease, so as to convey some idea of their nature and style: *

*He Kopito.*—Kopu nui, kopu roa, kopu takitaki, kopu whakaahu tena te ara te hamama na kawea kowhitia, pararitia, pupa, nau mai ki waho.

*Translation of the above Prayer for Swollen Stomach.*—Big belly, long belly, stretched belly, bursting belly. there is the passage open, take it hence, pluck it out.

*He Korere.*—Titi puru e, titi puru e, titi kohea, titi maiami, e tena te titi ka titi, tena te puru ka puru, ko te puru ra tena, i purua ai te tupuna a Houtaiki.

*Translation of the Prayer for Looseness of the Bowels.*—Stop up the looseness, stop up the looseness, the purging will subside, the purging be stayed: there is the purging and there is the stopping up, for this is the remedy that stayed the malady of thy ancestor Houtaiki.

*He Manawa.*—Kei te manawa, kei taku, kei taku manawa kei te manawa whena, he manawa kaukau. Tina ki roto whena ki roto whakataka atu ki roto.

*Translation of the above Prayer for Disease of the Heart, or Shortness of Breath.*—It is in my heart, on it is my breath, and in thy breath it is in thy heart, and my heart, in the heart that is strenuous. Let it be overpowered inside, let it be strenuous inside,† let it be thrust back inside.

*He Hono.*—Tutakino i ou iwi, tutakina i ou toto, tutakina i ou mengameya tena te rangi, ka tutaki, tena te papa ka whena.

*Translation of the above Prayer for a Sprained Back.*—Close up your bones, close up your blood, close up your marrow, and be united as the heavens, and let the bones be strong as the earth.

* I am indebted for these prayers, and for their translation, to Mr. C. O. Davis, of Auckland, one of the few persons in New Zealand intimately acquainted with the language and modes of thinking of the New Zealanders. The translations are literal, but it is a difficult subject, for few of the present generation of Maoris are acquainted with these prayers, or even the exact meaning of the words, and the difficulty is increased by the circumstance that many words have five or six meanings.

† This most probably refers to the god who is supposed to be in the heart of the sufferer.

Confidence was not, however, placed in such stupid compositions as the above, the exhibition of substances to cure diseases were occasionally resorted to; but the exact value of prayers and medicine will be at once appreciated when I state that medicine, without the assistance of prayers to the gods, was totally inefficacious. New Zealanders have no idea of the circulation of the blood, nor of any of the proper functions of the different organs of the body. The head, although extremely sacred, is not supposed to contain the organ which produces the intellect. The stomach and bowels are believed to be the seat of some of the faculties of the mind, such as joy, fear, and sorrow. Every external part of the body has a name, and a good many of the internal parts. Their cannibal feasts gave them an opportunity of acquiring anatomical knowledge.

The New Zealand mode of treating surgical cases was often successful and judicious. For a broken bone splints were made from the bark of trees in the shape of the part, and bandages were made from the flax plant. Dislocations were reduced. Sprains were treated with rest and shampooning. They never performed amputation. Scraped roots and leaves were applied hot for boils and sores. Abscesses and boils were opened. often long before they were ripe, and severe pressure applied to squeeze out the matter. The surgical instruments employed for opening abscesses were the sharp edge of a shell, a splinter of obsidean, or a sharp-pointed stick, or a thorn. If a person received a wound, it was first washed, and then a plaster of mud applied to exclude the air, and this was allowed to remain until the wound was well; sometimes the wound, if small, was bruised with a stone to excite bleeding, and afterwards held over the smoke of a fire of certain plants. The bleeding from a wound is sometimes checked by holding it over the smoke of a fire. For cutaneous diseases and sores, bathing in the hot sulphur and siliceous springs at Roturua Taupo, and at several other places, are reckoned very beneficial. For rheumatism scarifications were made in the skin, or friction and shampooning with the fat of pigeons and whale oil were used. In lumbago stones were heated and rolled over the part. Change from one part of the country to another was an esteemed remedy for certain diseases, not for the climate, but to avoid the evil spirits which produced the sickness. Blistering a part was known, and it was done by the application of the leaves of certain plants—e.g., clematis. Local bleeding by scarifying the skin was known, but not venesection. Hemorrhage from a wound was checked by bandages, and stuffing the wound with mud. Diseases which were particularly supposed to be caused from the devil, or an atua, living in the body, were occasionally starved out, *alias* the patient killed; at other times they were pressed out. Mr. Nicholas, a settler in the Thames, told me that in 1848

he saw a person killed by an attempt to drive out the atua. A young, healthy New Zealander had a pain in his stomach: he was laid out naked on the ground, heavy baskets of stones were placed on his chest and stomach, on the top of which several persons sat for about a quarter of an hour; when they were removed the unfortunate man was dead—from suffocation, I presume, in consequence of the movements of the lungs being impeded. The evil spirit, in this case, was effectually expelled, but with it went the vital spark.—*British and Foreign Medico-Chir. Journal.*

